# Extracellular vesicles release by cardiac telocytes: electron microscopy and electron tomography

**DOI:** 10.1111/jcmm.12436

**Published:** 2014-09-25

**Authors:** Emanuel T Fertig, Mihaela Gherghiceanu, Laurentiu M Popescu

**Affiliations:** a Electron Microscopy Laboratory, ‘Victor Babe’ National Institute of PathologyBucharest, Romania; b Department of Cellular and Molecular Medicine, ‘Carol Davila’ University of Medicine and PharmacyBucharest, Romania

**Keywords:** telocytes, heart, exosomes, ectosomes, multivesicular cargo, electron tomography, 3D reconstruction

## Abstract

Telocytes have been reported to play an important role in long-distance heterocellular communication in normal and diseased heart, both through direct contact (atypical junctions), as well as by releasing extracellular vesicles (EVs) which may act as paracrine mediators. Exosomes and ectosomes are the two main types of EVs, as classified by size and the mechanism of biogenesis. Using electron microscopy (EM) and electron tomography (ET) we have found that telocytes *in culture* release at least three types of EVs: exosomes (released from endosomes; 45 ± 8 nm), ectosomes (which bud directly from the plasma membrane; 128 ± 28 nm) and multivesicular cargos (MVC; 1 ± 0.4 μm), the latter containing tightly packaged endomembrane-bound vesicles (145 ± 35 nm). Electron tomography revealed that endomembrane vesicles are released into the extracellular space as a cargo enclosed by plasma membranes (estimated area of up to 3 μm^2^). This new type of EV, also released by telocytes *in tissue,* likely represents an essential component in the paracrine secretion of telocytes and may consequently be directly involved in heart physiology and regeneration.

## Introduction

Telocytes are a newly described type of interstitial cell, found in most organs and characterized by the presence of lengthy extensions (telopodes), visible by transmission electron microscopy (EM) 1 (www.telocytes.com and references cited therein). It has been reported that telocytes form a cardiac network that could integrate the overall ‘information’ from the vascular, nervous and immune systems, the interstitium, as well as stem cells/progenitors and working cardiomyocytes, by means of atypical junctions, nano-contacts and extracellular vesicles (EVs) 2. The presence of EVs in the proximity of telocytes has been shown by EM in normal 2,3 and diseased heart tissue 4, as well as other cavitary and non-cavitary organs 5–14.

Extracellular vesicles are membrane covered spheres that share the surface receptors and ligands of the parent cell and transport various biomolecules, such as miRNA 15, a large variety of proteins 16–18, even prions 19. This ability of EVs to function as intercellular cargos allow them to fulfil very complex roles in a variety of processes, ranging from cell-to-cell communication in the immune response and cancer progression, to angiogenesis and coagulation 20,21. EVs originating from telocytes were also shown to carry macromolecular signals (*e.g*. microRNAs) to neighbour cells 22, modifying their transcriptional activity 23 and to stimulate neo-angiogenesis in the border zone of infarcted myocardium 4.

Electron microscopy is currently the most reliable method for simultaneously classifying EVs based on their size, shape and density, while also allowing studies regarding their biogenesis 24–26. Exosomes are formed in the multivesicular endosome and secreted after its fusion with the plasma membrane, whereas ectosomes (also termed microvesicles) are formed by direct budding from the plasma membrane.

High resolution information of the morphology and 3D structure of EVs from telocytes obtained using ET may prove essential for better understanding their function as transporters of biomolecules and implicitly their role in intercellular communication. Here, we present a brief EM and ET analysis of EVs in interstitial cardiac cell cultures, revealing morphologically distinct multivesicular cargoes.

## Materials and methods

### Interstitial cell culture

The study was approved by the Bioethics Committee of the ‘Victor Babes’ National Institute of Pathology, Bucharest. Three month old Wistar rats were treated with 1000 U/kg heparin, then killed by cervical dislocation. The hearts were removed and placed in Hank's Balanced Salt Solution (HBSS) supplemented with 1% PSF (Penicilin, Streptomycin and Fungizone) and HEPES 0.01 mM, then dissected under the stereomicroscope and mechanically minced into ∼1 mm^3^ fragments. Enzymatic dissociation was done using 300 U/ml collagenase I in DMEM/F12 supplemented with 0.01 mM HEPES. The resulting solution was then filtered using a 40 μm strainer, then cells washed and resuspended in DMEM/F12 culture medium supplemented with 10% fetal calf serum, 100 U/ml penicillin and 100 mg/ml streptomycin. Cells were grown to confluence (80%) in 25 cm^2^ flasks, and then dislodged from the culture vessel with 2 mM EDTA at 37°C for 5 min. Finally, cells were re-plated on 35 mm Petri dishes for glutaraldehyde fixation. Unless otherwise specified, buffers and chemical reagents were supplied by Sigma-Aldrich Company Ltd., USA.

### Transmission electron microscopy

Cells were fixed with 2.5% glutaraldehyde in 0.1 M cacodylate buffer with 1.4% sucrose at pH 7.4 and 37°C, for 5 min. Cells were scraped, resuspended in the same fixative for 4 hr at 4°C and then post-fixed for 1 hr in buffered 1% OsO_4_ with 1.5% K_4_Fe(CN)_6_ (potassium ferrocyanide-reduced osmium). Fixed cells were spun at 850 × g, embedded in 1% agar gel, and further processed for final epoxy resin embedding (Agar 100). The epoxy resin was polymerized at 37°C for 48 hr. Thick sections (1 μm) were obtained using an MTXL RMC ultramicrotome (Boeckeler Instruments Inc., Arizona, USA) and then stained with 1% toluidine blue for visualization using light microscopy. The ultra-thin sections were cut using a diamond knife at 60 and 300 nm, double stained with 1% uranyl acetate and Reynolds lead citrate. The 60 nm thin sections were visualized using a Morgagni 268 TEM (FEI Company, Eindhoven, The Netherlands) at 80 kV. Digital electron micrographs were recorded with a MegaView III CCD and images processed using iTEM-SIS software (Olympus, Münster, Germany). All measurements were performed within the same software package.

### Electron tomography and 3D reconstruction

In the case of the 300 nm sections, goat anti-rabbit conjugated gold particles with a diameter of 10 nm (Aurion, Wageningen, The Netherlands) were used as fiducial markers. These were attached to both sides of the section by floating the grids on top of the gold-containing droplets, placed on a sheet of parafilm. Grids were then washed with ultrapure (Type 1) water to allow detachment of unbound particles and contaminants.

Electron tomography was performed at room temperature using a FEI Tecnai G2 Spirit TEM, equipped with a LaB_6_ cathode, at an acceleration voltage of 100 kV. Grid areas of interest were recorded by means of MegaView G2 CCD camera (Olympus) at nominal magnifications ranging from 21,000× to 60,000×. Single axis tilt series were collected using the Xplore3D (FEI) acquisition program, with final object sampling ranging from 2.6 to 4 nm/pixel. The angular tilt range was typically set from −60° to +60°, with increments of 1°. The tilt series images were aligned using the 10 nm gold fiducial markers visible on each section. Then, tomograms were generated from the aligned series using the eTOMO package. Finally 3D reconstructions were done by manually drawing coloured contours on each of the stacked images, using IMOD 4.5.3 software 27. When required, short animations were generated using the freely available video-editing software VideoMach (http://gromada.com/videomach/).

## Results

**Figure 1:**
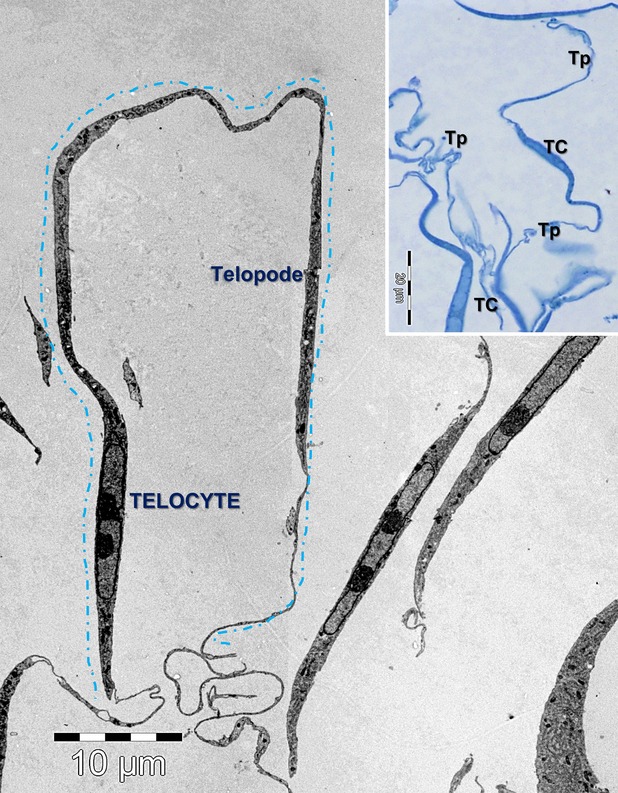
Electron microscopy image of interstitial cardiac cell culture shows a telocyte with about 100 µm long telopode. The inset shows a light microscopy image on blue-sections of telocytes (TC) *in culture* with long telopodes (Tp).

Telocytes were identified by EM in interstitial cardiac cell cultures based on the presence of long and thin cellular processes (telopodes) and a thin layer of cytoplasm surrounding the nucleus (Fig.1). The length of the telopodes, when measurable, ranged between 100 and 200 μm. The average width of telopodes was 114 ± 44 nm (mean ± SD, number of telopodes = 50), with a minimum width of 44 nm and a maximum of 406 nm.

**Figure 2:**
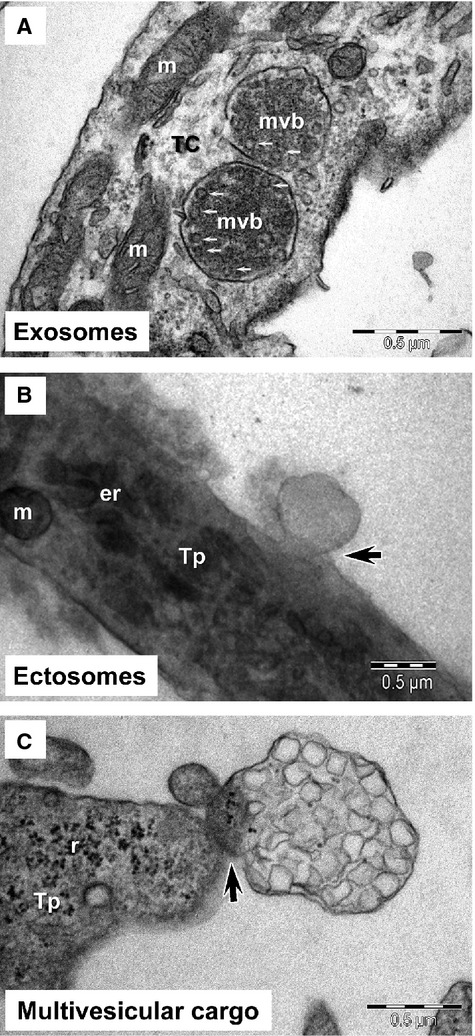
Electron microscopy of telocytes (TC) *in culture* demonstrates: (**A**) the presence of numerous intraluminal vesicles (small arrows) in two multivesicular bodies (mvb); (**B**) the ectosome budding (arrow) from plasma membrane of telopode (Tp); (**C**) a multivesicular cargo emerging (arrow) from a telopode (Tp). m, mitochondria; er, endoplasmic reticulum; r, ribosome.

Three distinct types of EVs were found in the proximity of the imaged cells (Fig.2): exosomes, imaged predominantly as intraluminal vesicles contained by multivesicular bodies (Fig2A), ectosomes budding from the plasma membrane (Fig.2B), as well as clusters of endomembrane vesicles enclosed by the plasma membrane (Fig.2C), a structure we termed ‘MVC’. Because of the preparation method used, which involved the centrifugation of cultured cells, the number of vesicles found in the vicinity of individual cells, could not be estimated.

Exosomes were not visible in the extracellular space most likely because of the embedding procedure employed. EM showed numerous multivesicular bodies containing intraluminal vesicles from which exosomes derive. The average diameter of intraluminal vesicles contained by multivesicular bodies was 45 ± 8 nm (mean ± SD, *n* = 50), with a minimum diameter of 33 nm and a maximum of 65 nm.

**Figure 3:**
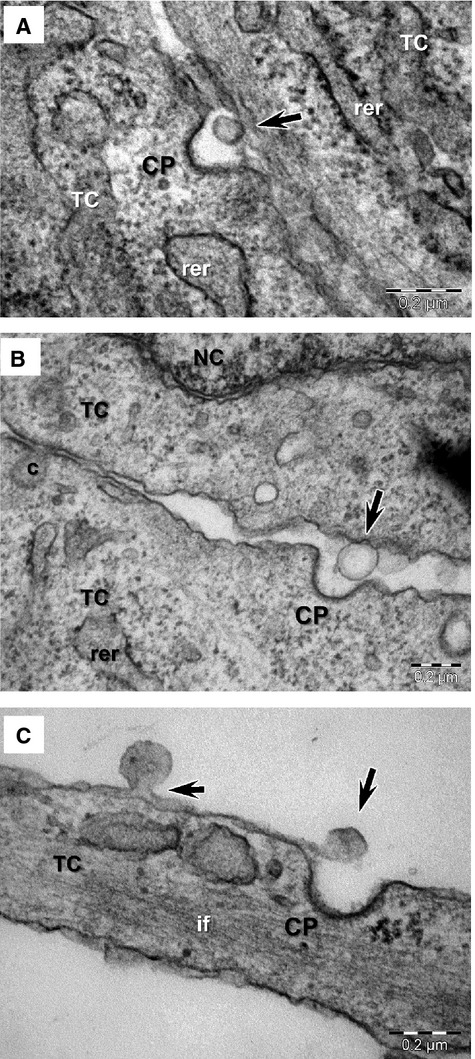
(**A**–**C**) Electron microscopy images of ectosomes released by telocytes (TC) *in culture* (arrows) frequently show the presence of clathrin coated pits (CP) in the vicinity. rer, rough endoplasmic reticulum; NC, nucleus; c, caveola; if, intermediate filaments.

Ectosomes were imaged budding directly from the plasma membrane of cultured cells, from both the cell body as well as from telopodes, consistent with the proposed mechanism of biogenesis 21. In contrast to the smaller exosomes, ectosomes had diameters between 70 and 165 nm, with an average of 128 ± 28 nm (mean ± SD, *n* = 25). Ectosomes were frequently observed in the vicinity of coated pits, which were identified based on their morphology (Figs2B, 3A and B). This suggests that the receptor-mediated endocytosis of these vesicles may be clathrin-dependent.

**Figure 4:**
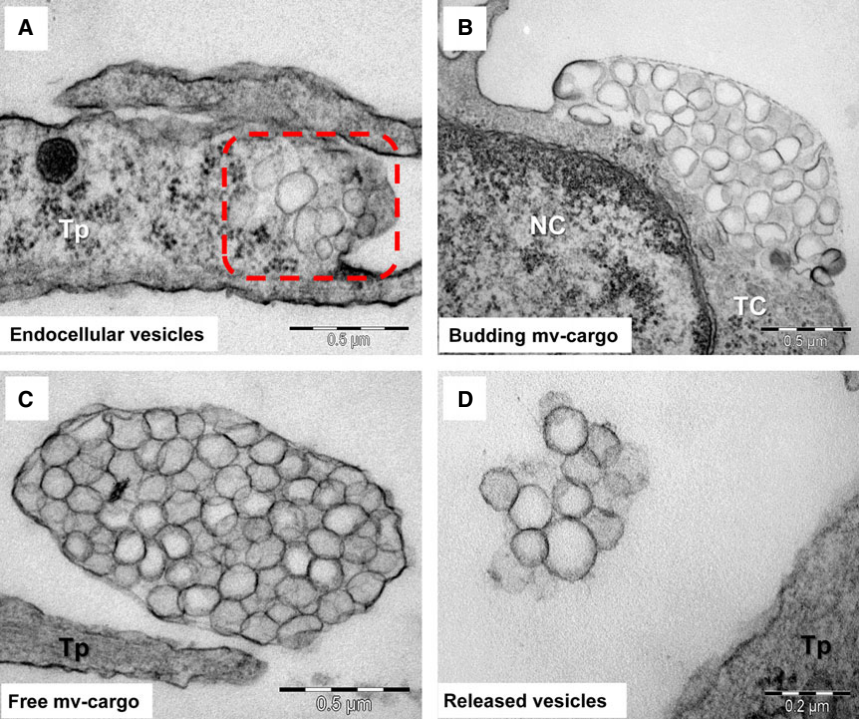
Possible mechanism of multivesicular cargo biogenesis based on electron microscopy images of telocytes (TC) *in culture*: (**A**) vesicles aggregation in the distinct space of cortical cytoplasm (square mark); (**B**) grouped endomembrane vesicles bulging a segment of plasma membrane; (**C**) gathered endomembrane-bound vesicles released into the extracellular space as a cargo shielded by plasma membrane; (**D**) dissolution of external membrane of multivesicular cargo release individual or grouped endomembrane vesicles into the extracellular space.

**Figure 5:**
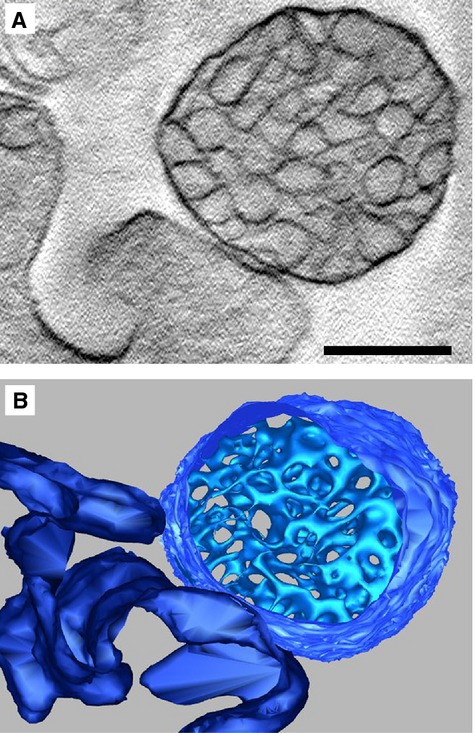
Electron tomography of multivesicular cargos (MVC): (**A**) Central tomogram section of a MVC, in direct contact with the cell membrane, revealing clustered vesicles enclosed by a membrane; (**B**) 3D model of section shown in (**A**) with isosurface representation of contained vesicles, revealing dense packaging. Cell membrane shown in dark blue. Scale bar represents 400 nm.

Notably, a third type of EV was also observed (Figs2C, 4 and 5). Endomembrane vesicles with an average diameter of 145 ± 35 nm (mean ± SD, 81÷206 nm, *n* = 50) were frequently imaged clustered in the cortical space of telocytes (Fig.4A), bulging the plasma membrane from both the cell body as well as telopodes (Fig.4B) and appearing to be released in an envelope formed by the plasma membrane (Fig.4C). The disruption of this envelope resulted in the release of the endomembrane vesicles into the extracellular space (Fig.4D). The MVCs had diameters between 0.4 and 1.5 micrometer, with an average value of 1 ± 0.4 μm (mean ± SD, *n* = 25), an estimated area ranging from 0.79 to 3.14 μm^2^ and estimated volumes of 0.05 to 0.52 μm^3^.

Additionally, MVCs were investigated for the first time by ET (Fig.5, Video S1). Consistent with EM studies, ET indicated a structure with a cup-shaped or ellipsoid morphology, containing anywhere between 60 and 500 tightly packed vesicles, of varying shapes and dimensions.

## Discussion

**Figure 6:**
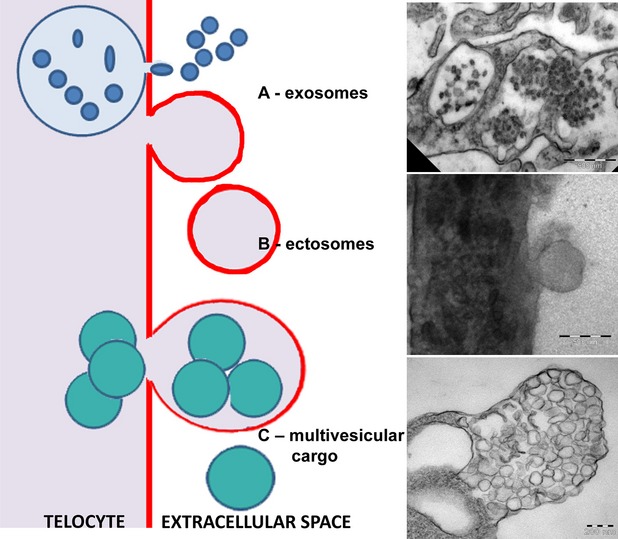
Schematic representation of the 3 types of extracellular vesicles released by telocytes and correspondent electron microscopy images of exosomes, ectosomes and multivesicular cargos.

Using EM and ET, we have found that telocytes *in culture* release at least three types of EVs: exosomes, ectosomes and multivesicular cargos enclosed by bi-layer membranes (Fig.6).

The latter have been previously described as matrix vesicles, specific to bone and cartilage and hypothesized to be involved in the tissue forming and remodelling processes 18 and misinterpreted as exosomes in mouse cardiac tissue 23. Here, we use EM and ET to characterize similar structures released by telocytes in interstitial cardiac cell cultures, suggesting their probably ubiquitous nature. We have termed these structures ‘MVC’ to emphasize their specific morphology and help distinguish them from other types of EVs. In our images MVCs are considerably larger than exosomes (50 and 100 nm, 20), whereas the reported dimensions of ectosomes are more similar (20–1000 nm, 20), allowing for an interval of overlap and making separation based on size alone difficult (*e.g*. differential centrifugation).

However, the mechanism of biogenesis for MVCs markedly differs from that of other EVs, such as exosomes and ectosomes, although ectosomes are also formed by budding of the plasma membrane. In the case of MVCs, clusters of endomembrane vesicles bulge the plasma membrane, by which they are gradually enveloped and then released into the extracellular space. Our observations suggest that after separating from the parent cell a disruption of the outer membrane occurs, allowing the release of the numerous clustered vesicles into the extracellular space. As in the case of ectosomes, the external plasma membrane envelope of MVCs may contain molecules important in target cell recognition. In contrast to individual ectosomes, however, MVCs may represent a more efficient method of dispersing biological signals to multiple target cells, as a single MVC could deliver up to 500 EVs.

The tight packaging of a high number of endomembrane vesicles within the multivesicular cargo was confirmed by ET. However, as a result of technical limitations associated with the preparation method used, higher resolution information regarding the structure and arrangement of these vesicles, could not be obtained. Future studies, potentially employing cryo-electron tomography may help elucidate whether the contained vesicles represent completely separate entities or if they form a network that allows molecules to relocate between vesicles.

To help elucidate the role that EVs play in heterocellular communication we are also employing biochemical methods. Specifically, we are currently investigating the contents of EVs released by intersitial cardiac cell cultures. For this purpose telocytes were transfected with miR21 mimic oligoRNA labelled with Cy5. The supernatant containing vesicles released by the miR transfected telocytes was collected and subsequently incubated with a different cell culture. Preliminary results show that recipient cells become Cy5 positive after incubation with the supernatant, indicating that telocytes release vesicles with miRs (data not shown). Additionally, previous studies have shown that a number of proteins preferentially expressed by telocytes may also be identified in EVs 28. Further investigation is required to determine the role that EVs play in telocyte-modulated tissue regeneration.

In conclusion, we have imaged and characterized clusters of small vesicles enclosed by endomembranes (multivesicular cargoes), released by cardiac telocytes in cell cultures. Furthermore, we have characterized these structures using ET for the first time, revealing the dense packaging of contained endomembrane vesicles.

## Supporting Information

Video S1 Electron tomography of a Multivesicular Cargo*.avi*.
