# Supplementary material for: Extracellular vesicles release by cardiac telocytes: electron microscopy and electron tomography
Source: J Cell Mol Med. 2014 Sep 25;18(10):1938–43. doi: 10.1111/jcmm.12436 (PMC4244009; doi:10.1111/jcmm.12436)
Supplement: Video S1 — Electron tomography of a Multivesicular Cargo.avi. [file jcmm0018-1938-sd1.docx]

Video S1
